# Biological traits approaches in benthic marine ecology: Dead ends and new paths

**DOI:** 10.1002/ece3.9001

**Published:** 2022-06-03

**Authors:** Silvia de Juan, Julie Bremner, Judi Hewitt, Anna Törnroos, Maria Cristina Mangano, Simon Thrush, Hilmar Hinz

**Affiliations:** ^1^ Instituto Mediterraneo de Estudios Avanzados IMEDEA (CSIC‐UIB) Esporles Islas Baleares Spain; ^2^ Centre for Environment Fisheries and Aquaculture Science Lowestoft England; ^3^ Collaborative Centre for Sustainable Use of the Seas University of East Anglia School of Environmental Science Norwich England; ^4^ Department of Statistics University of Auckland Auckland New Zealand; ^5^ Institute of Marine Science University of Auckland Auckland New Zealand; ^6^ Environmental and Marine Biology Faculty of Science and Engineering Åbo Akademi University Turku Finland; ^7^ Department of Integrative Marine Ecology (EMI) Stazione Zoologica Anton Dohrn Sicily Marine Centre Lungomare Cristoforo Colombo (complesso Roosevelt) Palermo Italy

**Keywords:** benthic communities, ecosystem function, effects traits, response traits, soft‐bottoms, traits‐based approaches

## Abstract

Biological traits analysis (BTA) links community structure to both ecological functions and response to environmental drivers through species’ attributes. In consequence, it has become a popular approach in marine benthic studies. However, BTA will reach a dead end if the scientific community does not acknowledge its current shortcomings and limitations: (a) uncertainties related to data origins and a lack of standardized reporting of trait information; (b) knowledge gaps on the role of multiple interacting traits on driving the organisms’ responses to environmental variability; (c) knowledge gaps regarding the mechanistic links between traits and functions; (d) a weak focus on the spatial and temporal variability that is inherent to the trait expression of species; and, last but not least, (e) the large reliance on expert knowledge due to an enormous knowledge gap on the basic ecology of many benthic species. BTA will only reach its full potential if the scientific community is able to standardize and unify the reporting and storage of traits data and reconsider the importance of baseline observational and experimental studies to fill knowledge gaps on the mechanistic links between biological traits, functions, and environmental variability. This challenge could be assisted by embracing new technological advances in marine monitoring, such as underwater camera technology and artificial intelligence, and making use of advanced statistical approaches that consider the interactive nature and spatio‐temporal variability of biological systems. The scientific community has to abandon some dead ends and explore new paths that will improve our understanding of individual species, traits, and the functioning of benthic ecosystems.

## Graphical abstract

The Biological traits analysis (BTA) is attractive as it is easily applied to past and present datasets, transcending the taxonomic composition of the study area and thus supporting inter‐regional comparisons; however, BTA is not exempt from challenges, and we propose solutions to bring us out of some dead ends and walk on new paths.

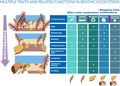

## 1 GENERAL INTRODUCTION

Ecosystem functioning is closely related to biological diversity, which has led to the development of trait‐based approaches to link community composition and functions (Díaz & Cabido, 2001; Loreau et al., 2001; Violle et al., 2007). Trait‐based approaches, in their broader sense, focus on the organisms’ attributes, ranging from single‐trait studies to multiple‐traits approaches (Kiørboe et al., 2018). Species’ attributes, or biological traits, are characteristics of an organism encompassing life history (e.g., life span), behavior (e.g., movement and migration, feeding ecology), and morphology (e.g., shape) (Bremner et al., 2003; Hewitt et al., 2008; Törnroos et al., 2015). Trait‐based approaches target the non‐taxonomic grouping of organisms that have similar traits and thus are expected to have similar influence on the environment (Gitay et al., 1999) or similar responses to environmental change (Gladstone‐Gallagher et al., 2019). Here, we focus on biological traits analysis (BTA) and its application to marine benthic studies; however, some of the issues raised within this paper also apply to other trait‐based analyses. BTA is a methodological approach that reduces the dimensionality of community assessments and seeks to find general rules for community ecology (McGill et al., 2006). The main property of BTA, within the wide array of trait‐based approaches, is that it considers the interacting matrix of traits derived from species, instead of the species themselves, which influence species–environment interactions and represent the functioning of the benthic system (Bremner et al., 2003).

**FIGURE 1:**
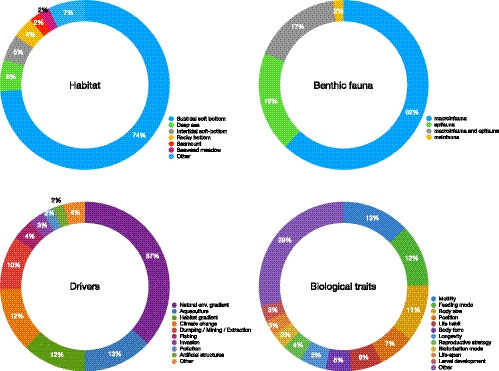
The literature on biological traits, as a summary of 90 published papers from 2003 to 2021 (Scopus literature database search in 25/12/2021), has widely covered marine habitats and benthic components, natural and anthropogenic drivers of change, and biological traits. The most commonly assessed drivers have been biotic and abiotic natural gradients, aquaculture, and climate change, whereas the most common traits have been the motility, feeding mode, body size, and environmental position

Since biologists first started to observe nature, there have been attempts to link species’ behavior and morphology with their interaction with the environment (Fauchald & Jumars, 1979; Rhoads & Young, 1971; Thorson, 1934; Woodin, 1978). Since the 1990s, the broader multifunctional trait‐based framework evolved in terrestrial and freshwater research (Diaz & Cabido, 1997; Lavorel et al., 2017) and, by the early 2000s, it became popular in marine studies. In a literature review undertaken to explore temporal trends and topics related with BTA publications in the marine benthos (details in Figure S1), a total of 168 publications were identified, starting with Bremner et al. (2003) and increasing by seven‐fold over the following decades, with the majority of studies focusing on benthic macrofauna living in subtidal soft‐bottom sediments (Figure 1). Equally, the review by Degen et al. (2018), on the general application of trait‐based approaches, confirmed this trend observing only 5% of marine studies including traits‐based approaches published before 2000, with the number of publications increasing three‐fold after 2010. In recent years, the initial emphasis on the link between biological traits and ecosystem functions has expanded to assess the recovery potential and resilience of benthic communities (e.g., Gladstone‐Gallagher et al., 2019; Hinz et al., 2021) as a way to advance our understanding of the implications of changes in community structure for the wider ecosystem.

The BTA approach is attractive as it is easily applied to past and present datasets, transcending the taxonomic composition of the study area and thus supporting inter‐regional comparisons (Beauchard et al., 2017; Bremner, 2008; Törnroos et al., 2015). However, BTA is not exempt from challenges and several limitations are the persistent gap (e.g., Degen et al., 2018; Lam‐Gordillo et al., 2020; Naeem & Bunker, 2009; Törnroos & Bonsdorff, 2012). The most critical weakness identified is the gap in the knowledge of species’ life history and behavior that is a clear limitation for the consolidation of BTA in marine ecology on a global scale. Another important limitation for advances in BTA research is the heterogeneity of trait nomenclature and inconsistent use of traits, and their scoring, across scientific studies. While trait selection is driven by research questions, some common definitions and guidelines are essential to allow for cross study comparisons and meta‐analysis to identify commonalities and draw sound conclusions. After two decades since its common adoption in benthic marine studies, BTA research needs to overcome these limitations if we want to further advance the field of marine functional ecology.

## 2 BTA IN MARINE SYSTEMS: ADVANTAGES AND CURRENT SHORTCOMINGS

The thriving publication of marine BTA research over the past two decades has allowed significant scientific advances, particularly in situations that have sought to contrast functional composition across habitats or environmental contexts (e.g., Bremner, 2008; Clare et al., 2015; Törnroos et al., 2015). The increase in BTA studies has partly been driven by its flexibility in different scenarios and data availability and popularity of the approach to assess functional properties of benthic systems (Beauchard et al., 2017). In practice, apart from application to existing large benthic datasets, BTA is often applied when complex sampling logistics or high costs hinder the collection of in situ measurements of community‐wide functional responses to environmental change (Muntadas et al., 2016) or to assess the sensitivity of benthic communities to environmental drivers (Hewitt et al., 2019). For example, collecting measures of bioturbation or nutrient fluxes alongside benthic community data implies a significant increase in logistic complexity and cost, and in many instances, it may not be feasible for regular temporal or large‐scale monitoring (but see Norkko et al., 2015). In these cases, ecosystem functions (e.g., bioturbation) have been estimated based on the multi‐trait composition of benthic communities (e.g., Hinz et al., 2021; Queirós et al., 2013; Solan et al., 2004).

Models that project ecosystem changes based on biological traits are becoming more popular. An example is the use of biological traits in biodiversity‐functioning models (e.g., Garcia et al., 2021; Kiørboe et al., 2018). Moreover, many scientific advice bodies are increasingly incorporating BTA approaches in monitoring protocols, and they are now being used to assess ecosystem‐wide effects of climate change and its links with ecosystem functions (Miatta et al., 2021). Despite its flexibility and advantages, the current BTA has several limitations and shortcomings which hinder its advance (see Sections 2.1–2.4). To examine these limitations, we discuss possible solutions (Section 3) that should facilitate bringing the BTA out of some dead ends and provide new paths (or revived old ones) to advance the field of functional benthic ecology.

### 2.1 Limitation 1: a reliance on expert knowledge and absence of a common set of criteria for trait selection

As many studies on marine benthos have to deal with upward of 100 species (e.g., Arvanitidis et al., 2009; Rees, 1999), it is impractical to expect to obtain empirical trait information for all species. Direct observation of benthic organisms’ behavior is a technological challenge in marine systems, and laboratory experiments often do not capture the complexity of species–environmental interactions (e.g., Needham et al., 2010, 2011), particularly at the seascape (Thrush et al., 2017). Therefore, the use of expert judgment is common in BTA approaches. Researchers’ expertise usually plays a role through the selection of trait data, for those species for which such information does not exist, from closely related species, belonging to the same genera or family. While this expert judgment provides flexibility for the BTA approach, it also introduces uncertainites and biases that are difficult to scale if the origin of the data is not specified. For example, species traits’ data often originate from a different region to the one studied, omitting the regional variability that may exist in trait expressions.

Expert knowledge plays a fundamental role in BTA studies, which confers many advantages to an approach that can be used in a range of data availability scenarios; but it also implies many challenges. Expert opinion needs to be clearly identifiable. Similarly, the foundation of such an expert opinion (e.g., whether traits are allocated based on similar species, morphological characteristics, or observer's experience) should be clearly stated in the data and resulting publications. Providing this information enables sensitivity assessments of the trait data, i.e., analysis of the effect of expert opinion/lack of data, to be more readily conducted. While Hewitt et al. (2011) and Thrush et al. (2017) detected no significant effect of different expert knowledge on the end results of BTA studies, this is likely to depend on the species composition of the community and the traits utilized. Benthic communities dominated by few species may be more prone to the bias of erroneous trait data compared to complex diverse communities (e.g., Muntadas et al. (2016) observed that in a heavily trawled fishing ground, a few dominant benthic species were driving traits’ composition). The effect of erroneous or uncertain data will depend on the affected traits and how closely they are linked to a specific function or response (e.g., Hinz et al. (2021) identified few species driving bioturbation).

There is also a need for scientific consensus on the use of common terms, as there is a great diversity in the language used for traits, hindering their general application and even leading to misinterpretation (Degen et al., 2018; Martini et al., 2021). The meaning of “trait” is simply a characteristic or attribute; the potential confusion appears when biological attributes are assumed to have a link with ecosystem processes, with no empirical basis, but are nevertheless wrongly named as “functional traits” (Box 1). Clarity of terminology is important, and it should be included in any scientific study. The potential solution is in having a high‐quality raw traits database to begin with that includes all the data in their original form; then, researchers can decide how to code (or not) the traits based on the question they want to answer. There have been many initiatives to create open‐access traits’ databases that can be consulted and that potentially can be updated and improved (see Table S1 for a list of existing open‐access data based on traits). Apart from these initiatives, traits data matrices are still scattered throughout publications by researchers or projects (see Table S2 for examples of traits commonly used in benthic studies). With no actual estimation of the role of expert knowledge, the generalization (or uptake) of information can be subjected to errors. The ideal scenario, besides an agreed data standard, would be a single global, or several regional, trait database(s), that would have a live character, nourished by continuous knowledge generation and exchange by the scientific community. Such initiatives would evidently also require long‐term backing by science funders.

BOX 1 **Definition of common terms used in BTA studies**

There is an abundance of terminology surrounding BTA, and it is often difficult to find precise definitions for these terms. In part, the terms overlap in their meaning or only have nuanced differences. To help the reader understand the differences, we have summarized their meaning as interpreted by the authors.

Ecological traits broadly encapsulate any morphological, physiological, or phenological feature measurable at the individual level, e.g., life history (e.g., life span or reproductive traits), behavior (e.g., movement and migration, feeding ecology), and morphology (e.g., shape) (Degen et al., 2018; Mcgill et al., 2006), often used interchangeably with “biological traits.”

Response traits are those traits related with the response of species to environmental factors, including disturbance, or the potential recovery of the species under more favorable conditions (Lavorel & Garnier, 2002). These traits are directly related to the survival of species, populations, or communities in changing environmental conditions. An example could be the effects of fecundity on the species’ recovery from disturbance, with high fecundity having positive effects on recovery.

Effect traits are those traits of an organism that contribute to an ecosystem function or process (Lavorel & Garnier, 2002). As such, the presence or activity of a species, depending on its abundance or biomass, body size, and metabolic rate, influences the functioning of the system to different degrees (e.g., bioturbation, habitat provision, filtration rate).

Functional traits describe the component of the phenotypic characteristics of an organism that influences ecosystem processes (Petchey & Gaston, 2006). This term incorporates ideas of both response and effect traits; however, it focuses more on the plasticity of traits at the level of individuals. A functional trait may only be described though direct measurements of individuals, e.g., size, body condition, size of maturity, movement speed, or ability to form habitat.

### 2.2 Limitation 2: overlooking the fact that benthic species and communities create an interacting matrix of biological traits that drive ecosystem functions and condition responses to environmental drivers

**FIGURE 2:**
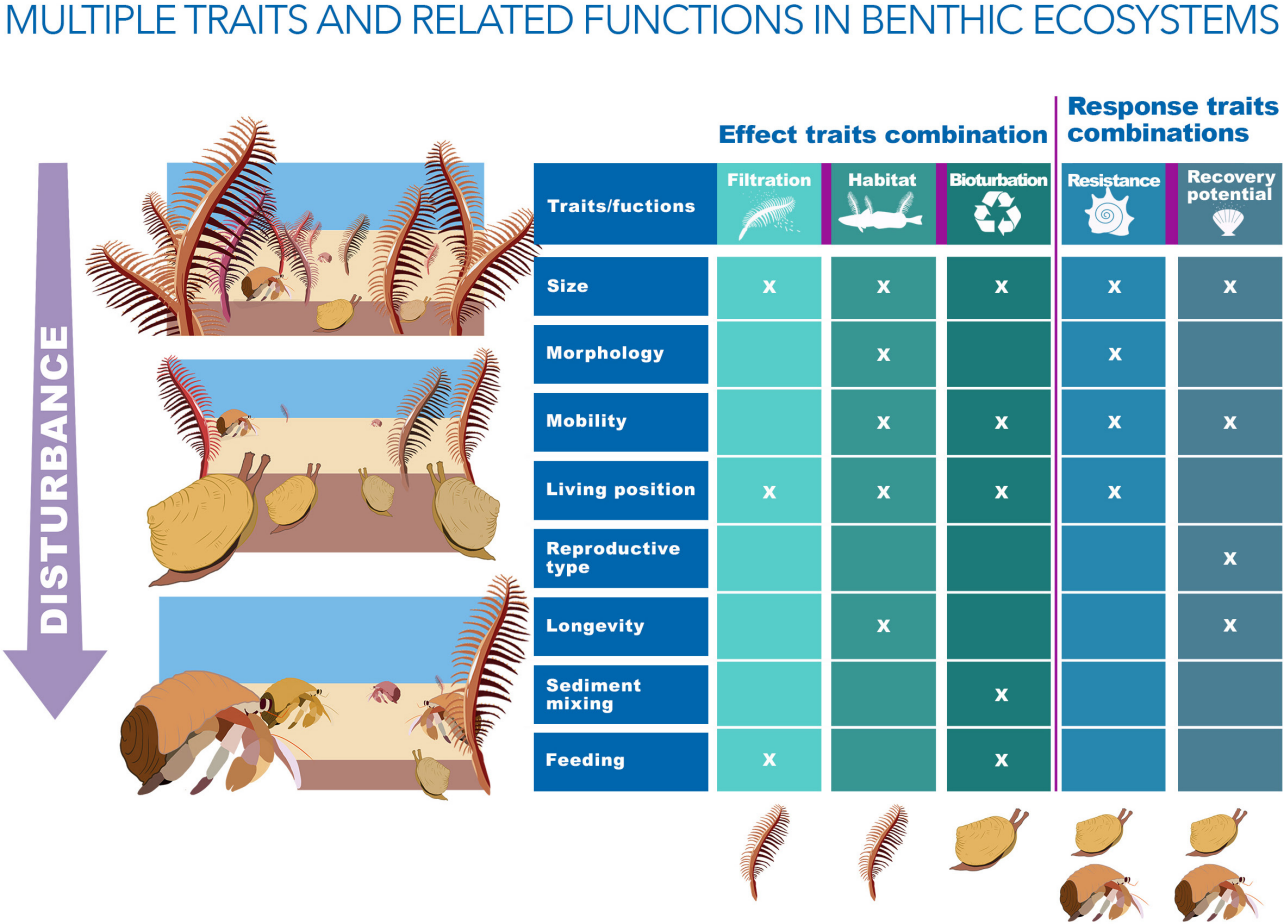
Example of a soft‐bottom benthic community (upper‐left corner) subjected to physical disturbance, such as trawling activities, that modify the community composition (bottom left corner). The combination of the organisms’ traits in the benthic community, such as size, morphology, mobility, and feeding drive the benthic community response to disturbance (in terms of resistance and recovery potential) and its potential to contribute to ecosystem functions, such as filtration (driven by large suspension feeders), habitat provision (driven by large sessile organisms), or bioturbation (driven by deposit feeders that move through the sediments creating bioturbation); the “x” in the table marks if a trait is included in the description of a particular function (Bremner, 2008; de Juan et al., 2007; Hinz et al., 2021). The organisms depicted below the columns of the functions are those that contribute disproportionally to that functionality. Note that the same organism can contribute to several functions. In case of the seapen, it contributes to both filtration and habitat provision, while providing less toward the bioturbation, the resistance, and the recovery potential of the community

The response of a species to a stressor depends on a certain combination of traits, some of which may be interacting with each other, and these interactions are not random. However, the interaction between traits does not always emerge from a BTA. Taking fishing disturbance as an example, generally, species living on the surface of the seabed are highly likely to be exposed to physical impact of the gear; however, if these species at the same time are of small size, mobile, and have a highly resistant shell, they may survive physical contact (de Juan et al., 2007). Based on the relative abundance of the organisms exhibiting the different traits, the “surface” trait might show positive responses to fishing disturbance. In this case, results need to be interpreted with knowledge on the species behind the traits; otherwise, the study might drive to erroneous conclusions on the benthic traits driving responses. Traits’ interaction might also be relevant for the link between traits and ecosystem function. In this case, the interaction between traits such as size, mobility, deposit feeding, and habitat might determine the contribution of a species to bioturbation. And the combination of these traits is very common in benthic communities: the type of mobility (e.g., from crawler to sedentary), the preferred position in the sediment (e.g., surface to deep burrowing), the deposit feeding mode (e.g., sediment uptake from surface to depth or from depth to surface), and the extent of sediment disturbance driven by the organism’ size. The interaction between individual traits can be established through combining them into new functional indices (Queirós et al., 2013; Solan et al., 2004). Within this approach, individual traits can thus be related to several ecosystem processes and functions, e.g., bioturbation, filtration, provision of habitat (Figure 2).

To establish a link between the species composition and the potential for ecosystem functions through the use of BTA, researchers need to define which set of interacting traits can be connected to functions to be able to assess what has changed in terms of functionality in different environmental variability scenarios (Figure 2). Currently, studies that have preselected traits related to a certain environmental driver or function (e.g., Hinz et al., 2021; Queirós et al., 2013; Solan et al., 2004) have done so through logical assumptions based on preexisting studies or expert knowledge about mechanistic links. To date, there are, to our knowledge, few studies that have attempted to empirically investigate and quantify these mechanistic linkages at scales relevant for benthic ecosystem processes (but see Douglas et al., 2017; Gammal et al., 2020; Norkko et al., 2015; O’Meara et al., 2020; Solan et al., 2004; Wrede et al., 2019). This may in part be related to the complexity of the task at hand, as it requires disentangling multi‐traits, multi‐species responses, and functions. However, expert knowledge should only be a starting point. Benthic ecologists should aim to increase efforts to demonstrate the mechanistic links between trait combinations and environmental variability and ecosystem functions. This research can be approached with analytical techniques that range from those used in marine studies for years (e.g., multiple regression and multivariate ordination) to techniques newer to the field with increasing computer power to quantify the relationship between multiple interacting elements of a system (e.g., network analysis, structural equation modeling).

### 2.3 Limitation 3: on the lack of detailed ecological baseline data

To evolve from an expert‐based multiple traits–functions matrix, a basic requirement is sound knowledge of trait–function relationships. To date, experiments that quantitatively explore specific trait–function relationships in the marine realm are quite rare (but see Lohrer et al., 2005; Michaud et al., 2005; Norkko et al., 2013; Thrush et al., 2017). In consequence, while for some common species data on mean trait values may be available, we have little knowledge of the variance of trait values or categories (e.g., the preference toward live prey vs. carrion for a predatory‐opportunistic organism), which is highly relevant for some functions. Trait categories often have wide arbitrary levels that reflect our lack of knowledge of the life history or behavior of a species. Movement is a simple example. Often, we classify mobility in broad categories such as sessile versus mobile, with no consideration of the wide range of possibilities between these two categories (e.g., limited mobility within a tube, burrowing, rafting/drifting, swimming or the actual measured or estimated movement range) and their relative contributions to resilience, recovery, or different ecosystem functions (e.g., bioturbation). Moreover, the increase in the detail in traits’ categories in the absence of verifiable empirical data might lead to a further reliance on expert knowledge as a data source that and introduce considerable uncertainties. With lack of knowledge on the effects of trait categories’ details in the study outcome, a balance between more detail and uncertainty must be achieved.

**FIGURE 3:**
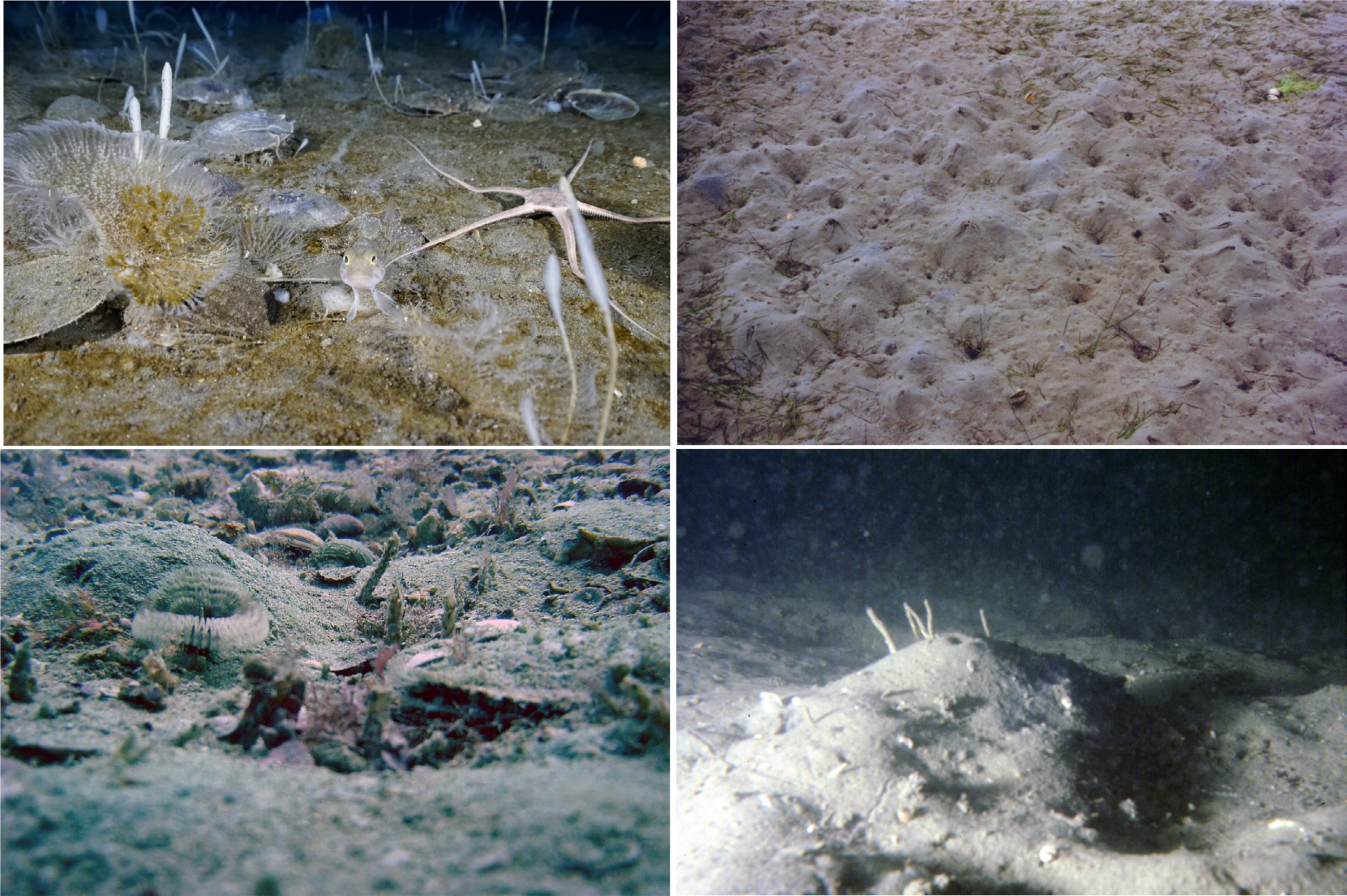
Images of soft‐bottom benthic communities evidencing emerging sediment structures and biogenic fauna that can indirectly inform of ecosystem functions. Starting upper‐left image and clockwise: (1) bivalve shells (*Adamussium colbecki*) provide primary settlement surfaces for sponges and Bryozoans (location: New Harbour, McMurdo Sound, Antarctica; PC: Peter (Chass) Marriott); (2) sediments highly bioturbated by ghost shrimps (*Biffarius filholi*) and (3) bioturbation mounts provide new habitats for tube worms (location: Otago Harbour, New Zealand; PC: Simon Thrush); (4) fan and tube worms are emergent fauna in bioturbated sediment with scallops and veneered bivalve shells (location: Queen Charlotte Sound, New Zealand; PC: Simon Thrush)

Baseline ecological studies on individual species have over the past decades lost their appeal to funders and scientists alike, being neither “value‐for‐money” or “sexy science.” However, this type of data collection is crucial to advance BTA and end its reliance on data of questionable origin and quality; baseline natural history information is the cornerstone of biodiversity science. Against the backdrop of new technologies that have become available, e.g., small sensors that can detect movement patterns, animal electronic tagging and the use of small inexpensive cameras, we now have the tools to collect this type of data in an efficient, precise, and cost‐effective way. Understanding the use of the sediment column then becomes possible, and we may be able to answer questions such as where do species live and feed and what effect do they have on the sedimentary environment. Furthermore, new methodologies may allow us to focus on the “landscape features” (Figure 3). For example, emerging sediment structures can be monitored to depict key functions and then relate to species’ traits producing them. New monitoring and sampling techniques associated with continuous recoding can place this in the temporal context (e.g., Coro & Bjerregaard Walsh, 2021; Hopkins et al., 2021).

### 2.4 Limitation 4: failure to integrate the spatio‐temporal variability inherent to benthic species in BTA

The introduction of new technologies for the monitoring of benthic species can also facilitate movement away from a rather static BTA and incorporate the habitat‐specific and life‐history variability on trait expression. Traditional BTA works on average traits for the species that are assigned based on our best knowledge on their biology and ecology. For example, most BTA use average or maximum adult size as the reference organism, which may hide the true variability within a dataset. Biological traits may change ontogenically (e.g., larval, post‐larval, and adult mobility), but also over shorter time scales (e.g., diurnal changes in activity) (e.g., Cassidy et al., 2020). Even if we assign species to different types of movement, how often during the day or year do these need to be exhibited? Many bivalves focus feeding during low‐flow periods, some deposit feeders only deposit feed at certain times and will suspension feed at others, and many small amphipods and crustaceans crawl/burrow during the day but at night will migrate up into the water column to travel long distances. While some traits might be largely static, e.g., morphology, other traits might be highly variable, e.g., burrowing behavior. The use of the average trait could bias predictions of both response to environmental drivers and effects on ecosystem functions. This variability linked to the environmental context needs to be understood in order to develop accurate models of biological trait‐ecosystem functioning.

There are two potential approaches to these problems. One is to search for another trait category that might explain the differences, e.g., ontogenetic differences in living habit are easily incorporated. Another approach is to allow for the incorporation of different types of data. For example, context variables (e.g., sediment type or current speed) could be analyzed utilizing statistical analyses that focus on non‐linearities and in detecting change points (e.g., by incorporation of regression trees). Plant ecology has much to share in terms of approaches for better elucidating intraspecific variability, with methods for comparing trait expression across the entirety of environmental gradients and for combining field observations with transplant experiments (“common garden experiments” in plant ecology terms) to tease apart genetic variation and trait plasticity (Ahrens et al., 2021; Anderegg et al., 2021). Vertebrate ecology also offers methodological insight, with researchers adapting functional diversity indices to incorporate intraspecific data (Manna et al., 2019) and statistical modeling of temporal multi‐population studies to disentangle ontogeny from environmental effects (Musseau et al., 2020). There are financial and logistical challenges in applying some of these methods to benthic ecology, where assemblages can be very diverse, physically remote, and species often rather small and difficult to study, but work in these other disciplines can guide further developments.

## 3 NEW PATHS: ON SOLUTIONS TO ADVANCE THE BTA APPROACH

There is a growing interest in incorporating BTA in ecological models and in regular monitoring programs that are especially crucial in the current biodiversity, climate, and sustainability crisis. Overcoming the current BTA limitations (Box 2) is therefore essential. In this context, we need to advance in collaborative science and build an improved and dynamic BTA by taking advantage of the positive aspects of the approach and proposing solutions to shortcomings.

BOX 2 **Summary of the future challenges for the advance of BTA**

1. Scientific studies applying BTA should routinely provide justification for the selection of traits based on mechanistic evidence and report the nature of trait data (expert knowledge, literature, empirical).
2. The BTA should incorporate a sensitivity analysis (including uncertainty in the input data), and a confidence assessment of the data sources each time BTA is used.

Points 1 and 2 should be the basic requirement for any BTA.

1. Researchers should explore new technologies, intelligent re‐use of survey outputs, non‐traditional data sources accompanied by a quality check (including citizen science initiatives), and the acquisition (and management) of big data to generate more baseline natural history data.
2. Benthic ecology needs to embrace traits’ variability (interspecific, intraspecific, environmental) beginning with the interpretation and discussion of results, but eventually incorporating variability in a multi‐dimensional species–traits matrices.

The current open‐access data initiatives can contribute to a more transparent and standardized naming, selection, and assignment of traits if they keep evolving with the required scientific needs. And to achieve this, scientists need to make a consolidated effort to reach a consensus for a unified data standard that would allow the exchange and validation of traits data. Such an initiative would not only facilitate data exchange between research groups but would also make it easier for scientists to update and contribute to these databases. Despite recent advances (see Table S1), the issues raised here are still largely unresolved. We suggest some minimum criteria for the reporting of traits data in future publications or entries in databases that would facilitate the use and integration of the reported data in the future: (i) a clear definition of the study objectives and a clear justification for the selection, inclusion, or exclusion of specific traits (e.g., response/effects traits); (ii) reporting of the source of each trait score, as either empirical, observational, or expert judgment, together with the appropriate citations (use of traits data from open‐access databases should be marked as such and extraction date noted when using live databases); (iii) include a data‐origin summary when interpreting traits data to help the reader understand the uncertainties that may be attached to the data; (iv) analyzing the potential effect of uncertainties. Open‐access databases should equally aim to publish the source of trait information provided. They need to go beyond the simple citation of scientific literature and provide a clear idea of the type of information provided (i.e., empirical, observational/anecdotal, or expert judgment). We propose a “live” database containing a glossary of biological traits for benthic communities specifying nature of the trait (e.g., effect, response, recovery). The key is in having a high‐quality raw traits database from which the researcher can decide how to code (or not) the traits based on the question they want to answer.

While BTA will necessarily rely on expert knowledge and the simplification of biological trait information by the categorization of traits, the research community needs to invest efforts in improving existing knowledge on the biology and functioning of benthic communities. Funding agencies will need to fund novel survey techniques, and examples can be found in fisheries science where the identification of fish has been automated with the help of artificial intelligence (Palmer et al., 2022). New data can be collected via innovative technology and methods development; for example, benthic sampling with vehicles such as AUVs/ROVs could record in situ morphological traits, size, position, body form, etc.; passive and active acoustic monitoring and sensors could be used to record movement rates; remotely piloted aircraft (i.e., drones) have potential for obtaining high‐resolution images of intertidal benthos (e.g., Chand et al., 2020; Hobley et al., 2021). Some of these technologies are still expensive, but low‐cost technologies are emerging and gaining traction. For example, cost‐effective video recording techniques offer a wide range of opportunities as well as continuous activity monitoring via accelerometer technology (Coro & Bjerregaard Walsh, 2021; Hopkins et al., 2021). On the other hand, the marine ecology community could be encouraged to record data on traits during routine studies, field courses, student teaching, etc. The development of protocols to collect traits information in regular surveys would support these initiatives. Citizen science, based on the collection of individual observations, is successful in generating large environmental datasets (Kelling et al., 2015; Ruiz‐Frau et al., 2020); the professional science community has the opportunity to do something similar, if funding and infrastructures to support the collection and processing of these records can be put in place.

In a parallel way, benthic ecologists should aim to increase efforts to demonstrate the mechanistic links between trait combinations and ecosystem functions under variable environmental scenarios. For example, there are a number of benthic species that can both suspension and deposit feed. Generally, they will swop if environmental conditions favor one feeding type (e.g., a suspension feeder might alter the feeding mode if suspended sediment concentrations are high) (Miller et al., 1992). The change in suspension/deposit feeding might be used to create robust categories of sensitivity to suspended sediment. For other traits, it may be the transfer from trait to function that changes with environmental constraints (e.g., living in a permanent burrow in mud while non‐permanent burrowing on sand). The inclusion of location/environmental information in the analysis might allow to change the probability of plastic species exhibiting certain traits and connecting to certain functions. While benthic ecologists continue to gather information on the species’ biology, the effects of traits’ combination on ecosystem functions can be approached relying on abiotic surrogates of species’ functions. This approach is increasingly valuable to fill knowledge gaps in the mechanistic link between a species’ characteristic and the functions it might perform; for example, sediment features are potential surrogates of seafloor animals’ activities. The utility of these landscape features might vary across functions, habitat types, and also across spatial and temporal scales. For example, faunal mediated mixing of the sediment (i.e., bioturbation) alters the structure of the sediment surface, and intense activities, e.g., paths, burrows etc. (Needham et al., 2011), can be visually identified and linked to the activity of benthic organisms. The measure of abiotic features facilitates upscaling of ecological studies at scales relevant to ecosystem functioning (Schenone et al., 2021). Computer Vision can be applied to automate the processing of vast amounts of information resulting from the remote and continuous acquisition of visual data in a cost‐effective and a high precision manner (Azhar et al., 2020; Martin‐Abadal et al., 2020).

Our proposal to use new technologies to monitor benthic communities and their environment in situ is also linked with the need to encompass temporal and spatial multidimensionality and dynamism. This would allow us to move forward from the traditional “snap‐shot” sampling of benthic communities toward continuous monitoring of the seabed that capture the variability in the exhibition of traits at short‐time scales or across space. The assessment of temporal dynamism in individuals’ sensitivity is an important future research topic (Hewitt et al., 2019). Additionally, the scaling up of functions required with ecosystem models needs to take into account the spatial variability in species–environment interactions and also species–species interactions that might imply a change in trait expression with different functional outcomes at the ecosystem level. To achieve this, benthic ecologists need to make use of contemporary computing power to exploit the multidimensionality of benthic communities (e.g., Siwicka et al., 2020, 2021; Siwicka & Thrush, 2020). Random forest classifiers (Kruk et al., 2017) could be explored to empirically grounded composite traits responses. Similar flexible Machine Learning techniques have been used to analyze and predict biological interactions between species (Pichler et al., 2020). Graph theory may also be good starting points for the integration of biophysical interactions (Miranda et al., 2013) into traits‐based approaches. While gaining a detailed understanding of how species change their expression of traits in various biotic and abiotic contexts may appear as an insurmountable task, we should aim to at least explore these for some key species. This would aid us in understanding the importance of these faceted interactions and mechanisms and may provide us with the potential for new analytical know‐how for the expansion of this type of investigation in the future.

The time is ripe to advance on BTA research and overcome its limitations, with growing international scientific collaboration, applying best practices, new technologies for data gathering, and ever‐increasing scientific knowledge on structure and dynamics of marine ecosystems and their key research questions. Moreover, large‐scale research on marine ecosystem functioning and its drivers is crucial to assess the consequences of global change and contribute to scientific knowledge needed to inform mitigation actions. In this context, BTA takes a crucial role in marine research and in the management of our shared seas.

## AUTHOR CONTRIBUTIONS

**Silvia de Juan:** Conceptualization (lead); Writing—original draft (lead); Writing—review & editing (lead). **Julie Bremner:** Conceptualization (supporting); Writing—original draft (equal); Writing—review & editing (equal). **Judi Hewitt:** Writing—original draft (equal); Writing—review & editing (equal). **Anna Törnroos:** Conceptualization (supporting); Writing—original draft (supporting); Writing—review & editing (equal). **Maria Cristina Mangano:** Writing—original draft (supporting); Writing—review & editing (supporting). **Simon Thrush:** Writing—original draft (supporting); Writing—review & editing (equal). **Hilmar Hinz:** Conceptualization (lead); Writing—original draft (equal); Writing—review & editing (equal).

## CONFLICT OF INTEREST

The authors declare no competing interests.

## Supporting information

Fig S1‐Table S1‐S2
