# Supplementary material for: Biological traits approaches in benthic marine ecology: Dead ends and new paths
Source: Ecol Evol. 2022 Jun 3;12(6):e9001. doi: 10.1002/ece3.9001 (PMC9163796; doi:10.1002/ece3.9001)
Supplement: Supplementary file 1 — Fig S1‐Table S1‐S2 [file ECE3-12-e9001-s001.pdf]

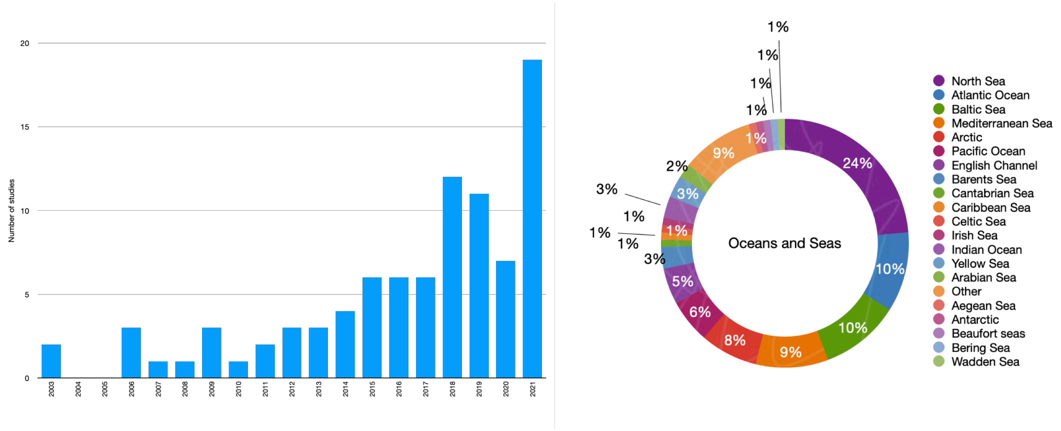

Figure S1. Evidence synthesis from the literature search. With the aim to search for scientific information on the study of biological traits, a complex search string was settled and ran on Scopus literature database (last run 25/12/2021). *Search string: TITLE-ABS-KEY (("biological trait*" OR "trait* based" OR "trait*-based") AND ("measure*" OR "experiment*" OR "analysis") AND ("marine" OR "sea") AND ("*benth*")).* A total of 168 scientific papers was retrieved. Scientific papers were screened per Title, Abstract and Full text in order to select only studies dealing with benthos and presenting a Biological Traits Analysis on marine ecosystems. From the papers that have passed the screening - 90 scientific papers - information on the temporal trend, the targeted oceans and seas, the target habitat and benthos fraction/dimension, the main analysed drivers of change and the main used biological traits were extracted, collated, and synthetised. The literature on biological traits, from 2003 to 2021, have widely covered oceans and seas (Other *1 = Aegean Sea 1%; Antarctic 1%; Beaufort Sea 1%; Bering Sea 1%; Cantabrian Sea 1%; Caribbean Sea 1%; Celtic Sea 1%; Irish Sea 1%; Wadden Sea 1%) and publications have increased over the past decade. In Figure 1 in the main text: Habitat “Other” = Canyons 1%; Coral reef 1%; Gravel and pebbles 1%; Intertidal rocky shore 1%; Mangrove 1%; Seagrass meadows 1%). Biological traits “Other” = Reproductive strategy 4%; Bioturbation mode 3%; Life-span 3%; Larval development 3%; Degree of attachment 2%; Sediments reworking 2%; Reproductive frequency 2%; Flexibility 2%; Larval type 2%; followed by Dispersal potential, Fragility, Egg development, Age at maturity, Sociability, Development mode, Body protection, Reproductive

season, Substratum affinity, AMBI index, Sexual differentiation, Trophic group (all individually represented by 1%) and by other 32 more traits all individually represented < 1%.

Table S1. Information on existing open access and/or access upon registration trait databases and/or trait compilations of marine taxa.

**Short name**	**Full name**	**Location (link)**	**Authors/reference to publication**	**Type of trait data and other info.**
EMODnetBiology	European marine observation and datat network - Biology	https://emodnet.ec.europa.eu/en/biology	Costello et al. 2015. Biological and ecological traits of marine species. PeerJ 3:e1201 https://doi.org/10.7717/peerj.1201	Data on temporal and spatial distribution of marine species and species traits from all European regional seas. Built upon the World Register of Marine Species (WoRMS)
BIOTIC	Biological trait information catalogue	http://www.marlin.ac.uk/biotic/	MarLIN, 2006. *BIOTIC - Biological Traits Information Catalogue.* Marine Life Information Network. Plymouth: Marine Biological Association of the United Kingdom. [Cited insert date]	Database contains information on over 40 biological trait categories on selected benthic species, with additional supporting information, including a bibliography of literature from which the information was obtained. The emphasis is on benthic invertebrates and plants.
Polytraits	A database on biological traits of polychaetes	http://polytraits.lifewatchgreece.eu	Faulwetter S. et al. (2014) *Polytraits*: A database on biological traits of marine polychaetes. *Biodiversity Data Journal* 2: e1024. doi:10.3897/BDJ.2.e1024	The database contains 47 different traits describing the morphological, behavioural, reproductive and larval features as well as the environmental affinities of a polychaetes (bristle worms, Polychaeta: Annelida)
FishBase	A Global Informaion System on Fishes	https://www.fishbase.se/search.php	-	FishBase is a global biodiversity information system on finfishes (but includes also some economically relevant invertebrate speceis). Contains taxonomic and biolgoical and ecological information of taxa.
Arctic traits	Arctic Traits database	https://www.univie.ac.at/arctictraits/	Degen R & Faulwetter S (2019) The Arctic Traits Database – a repository of Arctic benthic invertebrate traits, Earth System Science Data 11: 301-322. https://doi.org/10.5194/essd-11-301-2019	Focus on benthic invertebrates from Arctic regions. Currently 19 traits and 80 trait categories (morphology, life history, and behavior of species). Trait information per species is provided in text format (original literature source and quote provided) and in a 'fuzzy coded' mode (scores from 0 to 3). Taxon names are synchronized with WoRMS..
EOL	Encyclopedia of Life	https://eol.org	-	Includes biological and ecological information on a multitude of taxa
CESTES	A Global database for metacommunity Ecology: species, traits, environment and space	Information about the database: https://icestes.github.io	Jeliazkov et al. 2020. A global database for metacommunity ecology, integrating species, traits,	Collaction of 80 datasets from trait-based studies into a *global database for metaCommunity Ecology: Species, Traits, Environment and Space*; "CESTES". Each dataset includes four matrices: species

		Access to the database: https://idata.idiv.de/ddm/Data/ShowData/286	environment and space. *Scientific Data* **7**, 1-15.	community abundances or presences/absences across multiple sites, species trait information, environmental variables, spatial coordinates of the sampling sites.
-	Population dynamics in benthic invertebrates. A virtual handbook	http://www.thomas-brey.de/science/virtualhandbook	T. Brey, 2001. Population dynamics in benthic invertebrates. A virtual handbook. Version 01.2.	Conversion factors for units of matter and energy incl. Conversion factor data bank for aquatic species
sFDvent	sDiv-funded trait database for the Functional Diversity of vents	First version (sFDvent v.1) available through Supporting Information Table S4.1 in original publication: https://onlinelibrary.wiley.com/doi/10.1111/geb.12975#support-information-section	Chapman A.S.A et al. (2019). SFDvent: A global trait database for deep-sea hydrothermal-vent fauna. Global Ecology and Biogeography 28(11):1538-1551. https://doi.org/10.1111/geb.12975	Most known hydrothermal-vent species or taxa (in total 646) and scored species traits (in total 13.)
-	Coral Trait Database	https://coraltraits.org	Madin, J., Anderson, K., Andreasen, M. *et al.* The Coral Trait Database, a curated database of trait information for coral species from the global oceans. *Sci Data* **3,** 160017 (2016). https://doi.org/10.1038/sdata.2016.17	The Coral Trait Database is a growing compilation of scleractinian coral life history trait, phylogenetic and biogeographic data.
-	A trait database for marine copepods	https://doi.pangaea.de/10.1594/PANGAEA.862968	Brun P., Payne MR, Kiørboe T. (2016) A trait database for marine copepods. *PANGAEA*, https://doi.org/10.1594/PANGAEA.862968, Supplement to: Brun P. et al. (2017) A trait database for mairne copepods . Earth System Science Data, 9(1),99-113. https://doi.org/10.5194/essd-9-99-2017	Trait information on marine pelagic copepods from published literature and from experts, organized into a structured database. Includes 9306 records for 14 functional traits. Particular attention given to body size, feeding mode, egg size, spawning strategy, respiration rate, and myelination (presence of nerve sheathing).
SAMT	South Australian Macrobenthic Traits database	The database resource: https://doi.org/10.6084/m9.figshare.12763154<br><br>The SAMT v1.0.0 R package is currently available on the repository https://github.com/OrlandoLam/SAMT	Lam-Gordillo O., Baring R, Dittmann S. 2020 **https://doi.org/10.1002/ece3.7040**	The SAMT database includes 13 traits and 54 trait-modalities (e.g., life history, morphology, physiology, and behavior), and is based on records of macrobenthic fauna from South Australia. We provide trait information for more than 250 macrobenthic taxa, including outcomes from a fuzzy coding procedure, as well as an R package for using and analyzing the SAMT database.
AquaNIS	Information system on Aquatic Non-Indigenous and Cryptogenic Species	http://www.corpi.ku.lt/databases/index.php/aquanis/	AquaNIS. Editorial Board, 2015. Information system on Aquatic Non-Indigenous and Cryptogenic Species. World Wide Web electronic	AquaNIS is an online information system on the aquatic Non-Indigenous Species (NIS), and species which might be considered as NIS, i.e. cryptogenic species. The system stores and disseminates

			publication. Version 2.36+. Accessed 2021-30-11.	information on NIS introduction histories, recipient regions, taxonomy, biological traits, impacts, and other relevant documented data. Currently, the system contains data on NIS introduced to marine, brackish and coastal freshwater of Europe and neighboring regions
-	Fuzzy coding of biological traits of microbenthic species in the North Sea	https://epic.awi.de/id/eprint/37292/	Shojaei, M. , Brey, T. , Gutow, L. and Dannheim, J. (2013): Fuzzy coding of biological traits of macrobenthic species in the North Sea [Miscellaneous] doi: 10.1594/PANGAEA.813419	Data include seventeen biological traits related to life history (e.g. reproductive type) and behavior (e.g. feeding habit) of macrofuna species in the North Sea (fuzzy coding).
-	Bioturbation classification of European marine infaunal invertebrates	Available within the original publication: https://doi.org/10.1002/ece3.769	Queirós A.M. et al. (2013). A bioturbation classification of European marine infaunal invertebrates. Ecology and Evolution 3(11): 3958-3985. https://doi.org/10.1002/ece3.769	A compilation of functional classifications for 1033 benthic invertebrate species from the northwest European continental shelf for calculating Bioturbation potential.
-	Macrofauna trait collection from Finnish coastal areas, Baltic Sea.	Data collection available through supplementary information: https://doi.org/10.6084/m9.figshare.c.3295619.v1	Törnroos A., Bonsdorff E. (2012) Developing the multitrait concept for functional diveristy: lessons from a system rich in functions but poor in species. Ecologial Indicators, Vol. 22(8): 2221-2236. https://doi.org/10.1890/11-2042.1	Fuzzy coded trait information (25 traits in total) of marine zoobenthic taxa from the Baltic Sea, Finnish coastal regions.
-	Ten key biological traits of marine benthic invertebrates surveyed in Northwest Europe	https://data.cefas.co.uk/view/21362	Clare *et al*, Cefas (2022). Ten key biological traits of marine benthic invertebrates surveyed in Northwest Europe. Cefas, UK. V2. doi: https://doi.org/10.14466/CefasDataHub.123	This dataset contains information on ten key biological traits (behavioural, morphological, and reproductive characteristics) for over a thousand marine benthic invertebrate taxa surveyed in Northwest Europe (mainly the UK shelf). Scores of 0 to 3 are provided to indicate the level of confidence that taxa exhibit each possible mode of trait expression (0 = no evidence, 3 = strong evidence).

Table S2 – List of biological traits and categories commonly used in marine benthic studies by the authors of this work.

General trait	Trait category
Living position in sediment	Epibenthic (living on sediment surface) or protruding above
	Attached to other animals or small hard surfaces
	Top 2 cm
	Deeper than 2cm
	Living in crevices and under stones/shells or holdfasts
Adult movement/method	No movement
	Swimmer
	Rafter/Drifter/Byssus
	Crawler
	Burrower
Juvenile post-settlement movement/method	No movement
	Swimmer
	Crawler
	Rafter/Drifter/Byssus
	Burrower
Developmental type	Fragmentation
	Direct / brooders
	Lecithotrophic
	Planktotrophic
Feeding	Suspension feeder
	Deposit
	Predator
	Scavenger
	Grazer/herbivore
Creation of surface topography	Permanent burrow
	Simple hole or pit
	Tube
	Mound
	Trough- producing troughs in sediment
	Trampling across sediment surface
	no habitat structure
Living habit	Attached
	Tube/case dweller
	Burrow/crevice dweller- (not producing the burrow)
	Free
	Parasite/Commensal
	Arborescent, many branched
	Foliose, many branched but flexible
	Encrusting
	Erect singular
	Erect forming beds
Sediment moving/mixing by animal movement	Surface (top 2 cm)-to-deep (>2cm deep)
	Deep-to-surface
	Surface mixing

	Deep mixing
	no sediment mixing
Adult size	0-10 mm longest dimension
	11-20 mm
	21-50 mm
	51-100 mm
	>100 mm
Adult longevity	<1 yr
	1-3 yrs
	3-6 yrs
	6-10 yrs
	>10 yrs
Form	Vermiform length >>> width (at least 5 X)
	Streamlined length >>width (at least 2 X)
	Globulose, length ~ width
Reproductive frequency	Semelparous
	Iteroparous
	Semi-continuous
Rigidity	predominantly soft-bodied or flexible
	rigid, animals with exoskeletons, some echinoderms, animals with rigid tube structures (e.g. owenids and some maldanids, not polydorids
	Contains calcium carbonate
